# Tenecteplase vs. alteplase for treatment of acute ischemic stroke: A systematic review and meta-analysis of randomized trials

**DOI:** 10.3389/fneur.2023.1102463

**Published:** 2023-01-23

**Authors:** Hassan Kobeissi, Sherief Ghozy, Bilal Turfe, Cem Bilgin, Ramanathan Kadirvel, David F. Kallmes, Waleed Brinjikji, Alejandro A. Rabinstein

**Affiliations:** ^1^Department of Radiology, Mayo Clinic, Rochester, MN, United States; ^2^School of Medicine, Ross University, Bridgetown, Barbados; ^3^Department of Neurologic Surgery, Mayo Clinic, Rochester, MN, United States; ^4^Department of Neurology, Mayo Clinic, Rochester, MN, United States

**Keywords:** tenecteplase, alteplase, stroke, meta-analysis, ischemic stroke, TNK, TPA

## Abstract

**Background and objectives:**

Several randomized controlled trials (RCTs) have compared tenecteplase to alteplase for treatment of acute ischemic stroke (AIS). Yet, there is no meta-analysis that includes the latest published RCTs of 2022. We sought to compare the safety and efficacy of tenecteplase vs. alteplase for the treatment of AIS through a meta-analysis of all published RCTs.

**Methods:**

A systematic literature review of the English language literature was conducted using PubMed, Web of Science, Scopus, and Embase. We included RCTs that focused on patients with AIS treated with tenecteplase and alteplase. Multiple reviewers screened through potential studies to identify the final papers included in our analysis. Following PRISMA guidelines, multiple authors extracted data to ensure accuracy. Data were pooled using a random-effects model.

**Results:**

Nine trials, with 3,706 patients, compared outcomes of patients treated with tenecteplase and alteplase for AIS. Both treatments resulted in comparable rates of modified Rankin Scale (mRS) 0–1 at 90 days (RR = 1.03; 95% CI = 0.97–1.10; *P*-value = 0.359) and mRS 0–2 at 90 days (RR = 1.03; 95% CI = 0.87–1.22; *P*-value = 0.749). There was no heterogeneity among included studies regarding mRS 0–1 rates (I^2^ = 26%; *P*-value = 0.211); however, there was significant heterogeneity in mRS 0–2 rates (I^2^ = 71%; *P*-value = 0.002). Similarly, rates of mortality (RR = 0.97; 95% CI = 0.81–1.16; *P*-value = 0.746) and symptomatic intracranial hemorrhage (sICH) rates (RR = 1.10; 95% CI = 0.75–1.61; *P*-value = 0.622) were comparable in both treatment groups. There was no significant heterogeneity among included studies in either mortality (I^2^ = 30%; *P*-value = 0.181) or sICH (I^2^ = 0%; *P*-value = 0.734) rates. Further analysis comparing dosing of tenecteplase (0.1, 0.25, 0.32, and 0.4 mg/kg) yielded no significant differences for any of the endpoints (mRS 0–1, mRS 0–2, sICH, and mortality) compared to alteplase.

**Discussion:**

Based on available evidence from completed RCTs, tenecteplase has proven similar safety and efficacy to alteplase for treatment of AIS.

## Introduction

Tenecteplase, a genetically modified variant of alteplase, is being increasingly used for treatment of acute ischemic stroke (AIS). Although alteplase remains the only approved thrombolytic for treatment of AIS in the United States, tenecteplase offers theoretical and practical advantages when compared to alteplase (1). Because of its longer half-life (22 vs. 4 min for alteplase) (2), tenecteplase can be administered as a single bolus, a major practical advantage particularly for patients who require transportation to another center. Also, as compared to alteplase, tenecteplase has a 15-fold higher specificity for fibrin and an 80-fold decreased binding affinity to plasminogen activator inhibitor-1 (PAI-1) (2).

Tenecteplase is the thrombolytic of choice for patients with acute ST-elevation myocardial infarction (3). Yet, despite multiple RCTs and prospective studies comparing tenecteplase to alteplase for AIS, tenecteplase is not yet approved for the treatment of AIS in the United States, although other countries have approved its use (4). Haley et al. published the first RCT comparing the two thrombolytics in 2010, demonstrating the potential role of tenecteplase for AIS (5). Since then, eight more RCTs have been published comparing tenecteplase to alteplase, with three trials being published in 2022 alone (6–13). These RCTs have not yet been pooled together into a meta-analysis.

To assess the present evidence comparing tenecteplase and alteplase, we conducted a systematic review and meta-analysis of RCTs that reported clinical and safety outcomes following thrombolytic treatment for AIS.

## Methods

### Search strategy

On 7th September 2022, following the Preferred Reporting Items for Systematic Reviews and Meta-Analyses (PRISMA) 2020 guidelines for performing systematic reviews, a systematic literature review of the English language literature was conducted within the Nested Knowledge Autolit software per the drafted protocol, from inception, using PubMed, Embase, Web of science, and Scopus (14). Based on each database, different combinations of possible keywords and/or MeSH terms were used for that purpose. Keywords and MeSH terms included: “tenecteplase” AND “stroke”. Moreover, we did an extensive manual search through the references of the included articles to retrieve any missed papers.

### Screening process

We included all original studies fulfilling our pre-determined PICO. Population was patients with AIS, Intervention treatment with tenecteplase, Control group treatment with alteplase, the Outcomes of interest were the modified Rankin Scale (mRS) 0–1, mRS 0–2, mortality and symptomatic intracranial hemorrhage (sICH). We excluded papers where patients did not have AIS, were not treated with tenecteplase and alteplase, review articles, duplicate studies including the same patients presented in other included paper, case reports, case series with < 5 patients, and conference abstracts. We included RCTs and excluded all other study designs.

Two authors performed the title and abstract screening against the pre-defined criteria. This was followed by a full text screening of any retained studies of the first screening step. In both stages, the senior author was consulted to resolve any conflicts in the decisions.

### Data extraction

Following a pilot extraction, an extraction sheet was built, and the extraction was performed by at least two authors. The extracted data included study characteristics, baseline data of the included patients, and the aforementioned outcomes of interest. After performing the extraction, a third author performed an extensive revision of the extracted data to avoid any prior mistakes.

### Risk of bias

The “Cochrane RoB 2: a reivsed tool for assessing risk of bias in randomized trials” was used to assess the risk of bias, with two independent reviewers evaluating all studies (15). Two authors evaluated the quality of each study and adjudicated by a third one, whenever needed.

### Statistical analysis

Using the package “meta”, we conducted a pairwise meta-analysis to compare tenecteplase (any dose) and alteplase. Because < 10 studies were included in our analysis, assessing publication bias using Egger's regression test was not possible. In presence of double zero events (sICH outcome), we used Haldane's continuity correction (16, 17). For further insights, we used the “netmeta” package to conduct a frequentist network meta-analysis to compare different tenecteplase doses (0.1, 0.25, 0.32, and 0.4 mg/kg) and alteplase. A random-effects model was used to perform the network meta-analysis due to methodological heterogeneity contradicting with the common effect assumption. The pooled risk ratios (RRs) were considered heterogenous whenever I^2^ was higher than 50% and/or *p*-value < 0.05, as assessed by Q-statistics. Treatments ranking was based on *P*-scores, which are the frequentist approach analog to surface under the cumulative ranking (SUCRA) (18). Whenever ten or more comparisons were pooled for an outcome, comparison-adjusted funnel plots were built to examine the risk of bias and small-study effects (19). Funnel plot asymmetry was assessed with three different tests; Egger's regression, Begg-Mazumdar, and Thompson-Sharp tests with *P*-value < 0.05 were considered significant (20–22). All data were analyzed using R software version 4.2.1.

### Data availability

The data that support the findings of this study are available from the corresponding author on reasonable request.

## Results

### Search and screening results

Following the removal of 684 duplicate records, we retrieved 879 papers for further screening. Moreover, we excluded 870 records through the title and abstract screening stage, to retain nine records for full-text screening. Finally, nine papers were determined to satisfy our inclusion criteria with the appropriate report of outcomes of interest (Supplementary Figure 1).

### Study characteristics and risk of bias

All nine studies included in our analysis were RCTs. The size of the included studies ranged from 75 patients to 1,577 patients. All nine RCTs included in our analysis were deemed to have a “low” risk of bias (Supplementary Table 1, Supplementary Figure 2). Study characteristics, such as age, baseline NIHSS, dosage of tenecteplase, time window for treatment, and co-morbidities are detailed in Table 1.

**Table 1:** Characteristics of the included studies.

	**Haley et al**.	**Parsons et al**.	**Huang et al**.	**Logallo et al**.	**Campbell et al**.	**Li et al**.	**Bivard et al**.	**Menon et al**.	**Kvistad et al**.
**Year**	**2010**	**2012**	**2015**	**2017**	**2018**	**2021**	**2022**	**2022**	**2022**
Countries	United States	Australia	Scotland	Norway	Australia and New Zealand	China	Australia	Canada	Norway
Patients, *n*	112	75	96	1,100	202	236	104	1,577	204
TNK dose (s), mg/kg	0.1/0.25/0.4	0.1/0.25	0.25	0.4	0.25	0.1/0.25/0.32	0.25	0.25	0.4
Age, years, mean (SD) or median (IQR)	TNK 0.1: 67 (19); TNK 0.25: 69 (15); TNK 0.4: 68 (16); tPA: 72 (16)	TNK 0.1: 72 (6.9); TNK 0.25: 68 (9.4); tPA: 70 (8.4)	TNK: 71 (12); tPA: 71 (13)	TNK: 70.8 (14.4); tPA: 71.2 (13.2)	TNK: 70.4 (15.1); tPA: 71.9 (13.7)	TNK 0.1: 62.4 (11.1); TNK 0.25: 64.3 (12.8); TNK 0.32: 64.8 (12.1); tPA: 66.5 (12.6)	TNK: 76 (60–84); tPA: 73 (61–80)	TNK: 74 (63–83); tPA: 73 (62–83)	TNK: 73.2 (12.6); tPA: 68.6 (15.6)
Sex, male	58 (51.8%)	38 (50.7%)	61 (63.5%)	660 (60%)	110 (54.5%)	170 (72%)	63 (60.6%)	822 (52.1%)	98 (48.0%)
Severity (NIHSS), mean (SD) or median (IQR)	TNK 0.1: 8 (5–11); TNK 0.25: 10 (6–15); TNK 0.4: 9 (5–17); tPA 13 (5–17)	TNK 0.1: 14.5 (2.3); TNK 0.25: 14.6 (2.3); tPA: 14.0 (2.3)	TNK: 12 (9–18); tPA: 11 (8–16)	TNK: 5.6 (5.4); tPA: 5.8 (5.2)	TNK: 17 (12–22); tPA: 17 (12–22)	TNK 0.1: 7 (5–10); TNK 0.25: 8 (5–12); TNK 0.32: 7.5 (6–12); tPA: 8 (5–12)	TNK: 8 (5–14); tPA: 8 (5–17)	TNK: 9 (6–16); tPA: 10 (6–17)	TNK: 13.4 (6.6); tPA: 13.2 (6.4)
Permitted time window	< 3 h	< 6 h	< 4.5 h	< 4.5 h	< 4.5 h	< 3 h	< 4.5 h	< 4.5 h	< 4.5 h
Onset to treatment, min, median (IQR) or mean (SD)	–	Overall: 174 (48); TNK 0.1: 186 (54); TNK 0.25: 180 (42) tPA: 162 (48)	TNK: 180 (156–215); tPA: 200 (160–220)	TNK: 118 (79–180); tPA: 111 (80–174)	TNK: 125 (102–156); tPA: 134 (104–176)	TNK 0.1: 154 (56–195); TNK 0.25: 149 (80–179); TNK 0.32: 147 (69–220); tPA: 153 (18–187)	TNK: 45 (34–66); tPA: 50 (32–69)	TNK: 128 (93–186); tPA: 131 (95–188)	TNK: 92.5 (74–143); tPA: 99 (73–143)
Atrial fibrillation	–	28 (37.3%)	34 (35.4%)	119 (10.8%)	–	32 (13.6%)	15 (14.6%)	–	17 (8.3%)
Hypertension	89 (79.5%)	47 (62.7%)	48 (50%)	482 (43.8%)	–	157 (66.5%)	61 (58.7%)	–	104 (51.0%)
Dyslipidemia	56 (50%)	37 (49.3%)	11 (11.5%)	126 (11.5%)	–	51 (21.6%)	43 (41.3%)	–	63 (30.9%)
Diabetes mellitus	21 (18.8%)	15 (20.0%)	14 (14.6%)	144 (13.1%)	–	49 (20.8%)	28 (27.2%)	–	28 (13.7%)
Current smoker	16 (14.2%)	15 (20.0%)	23 (24.0%)	346 (31.5%)	–	95 (40.2%)	17 (16.5%)	–	49 (24%)
sICH definition	NINDS study	SITS-MOST	ECASS II	ECASS III	SITS-MOST	ECASS III	SITS-MOST	SITS-MOST	ECASS III

TNK, tenecteplase; tPA, alteplase; SD, standard deviation; IQR, interquartile range; NIHSS, National Institute of Health Stroke Scale; sICH, symptomatic intracranial hemorrhage.

### Tenecteplase vs. alteplase

**Figure 1:**
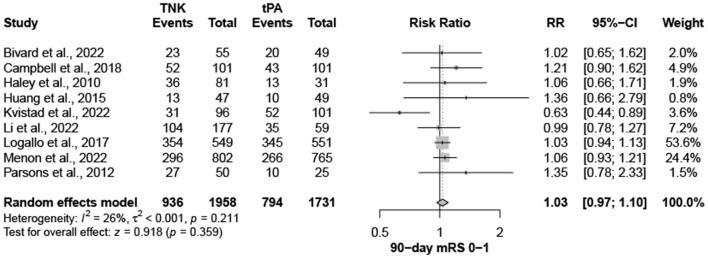
Rates of mRS 0–1 at 90 days. TNK, tenecteplase; tPA, alteplase.

**Figure 2:**
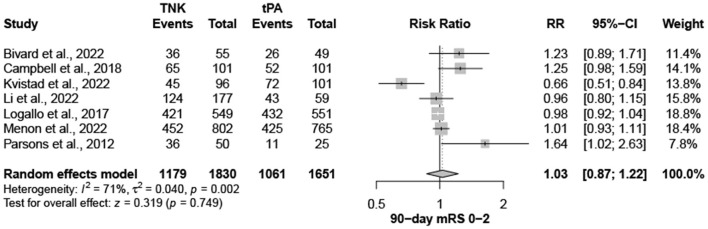
Rates of mRS 0–2 at 90 days. TNK, tenecteplase; tPA, alteplase.

**Figure 3:**
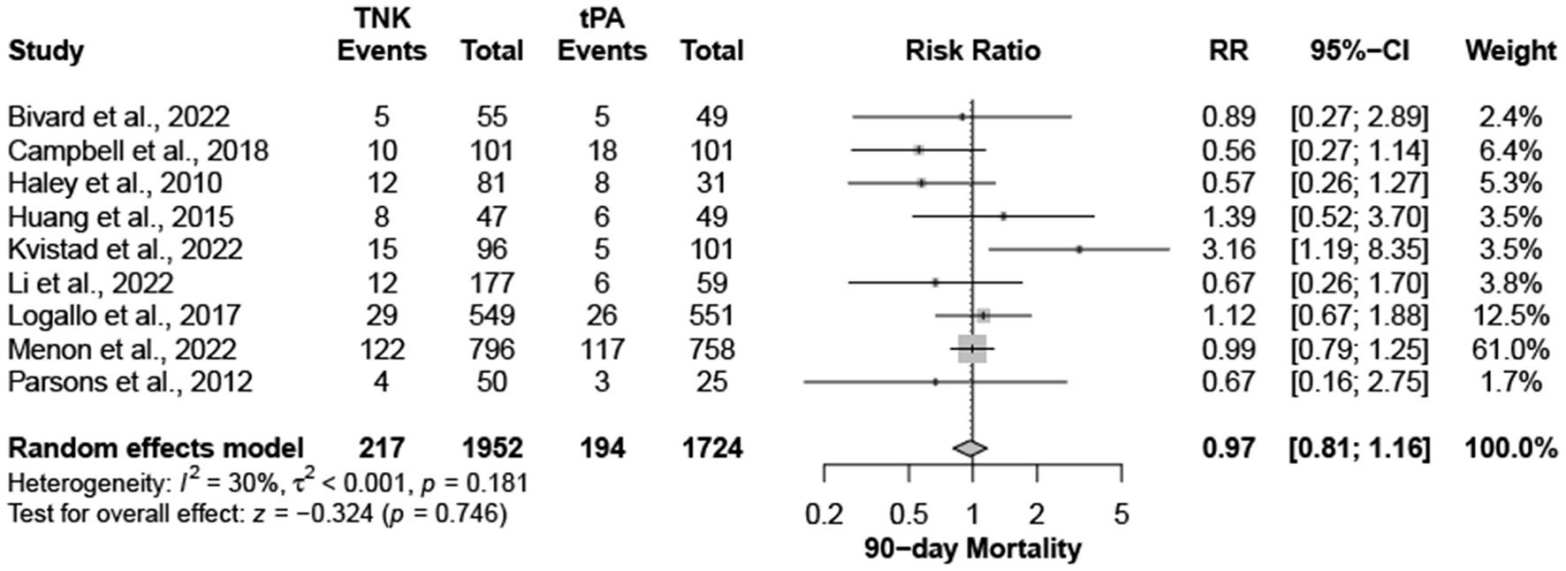
Rates of mortality at 90 days. TNK, tenecteplase; tPA, alteplase.

**Figure 4:**
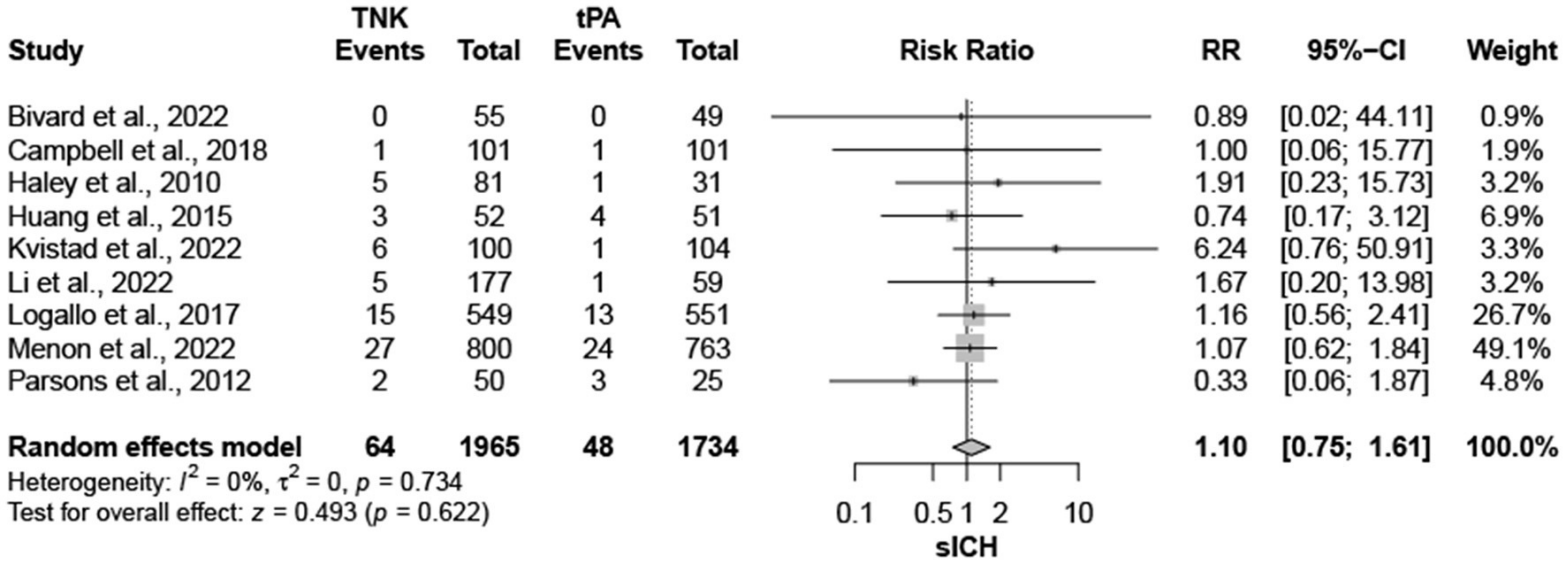
Rates of sICH. TNK, tenecteplase; tPA, alteplase.

Nine trials, with 3,706 patients, compared outcomes of patients treated with tenecteplase and alteplase for AIS. Both treatments showed comparable rates of mRS 0–1 (RR = 1.03; 95% CI = 0.97–1.10; *P*-value = 0.359) and mRS 0–2 (RR = 1.03; 95% CI = 0.87–1.22; *P*-value = 0.749) at 90 days. There was no significant heterogeneity among included studies regarding mRS 0–1 rates (I^2^ = 26%; *P*-value = 0.211) (Figure 1); however, there was significant heterogeneity for mRS 0–2 rates (I^2^ = 71%; *P*-value = 0.002) (Figure 2). Similarly, mortality rates (RR = 0.97; 95% CI = 0.81–1.16; *P*-value = 0.746) and sICH rates (RR = 1.10; 95% CI = 0.75–1.61; *P*-value = 0.622) were comparable in both treatment groups (Figure 3). There was no significant heterogeneity among included studies in either mortality (I^2^ = 30%; *P*-value = 0.181) or sICH (I^2^ = 0%; *P*-value = 0.734) rates (Figure 4). The definitions of sICH for each included RCT can be found in Table 1.

### Different tenecteplase doses vs. alteplase

**Figure 5:**
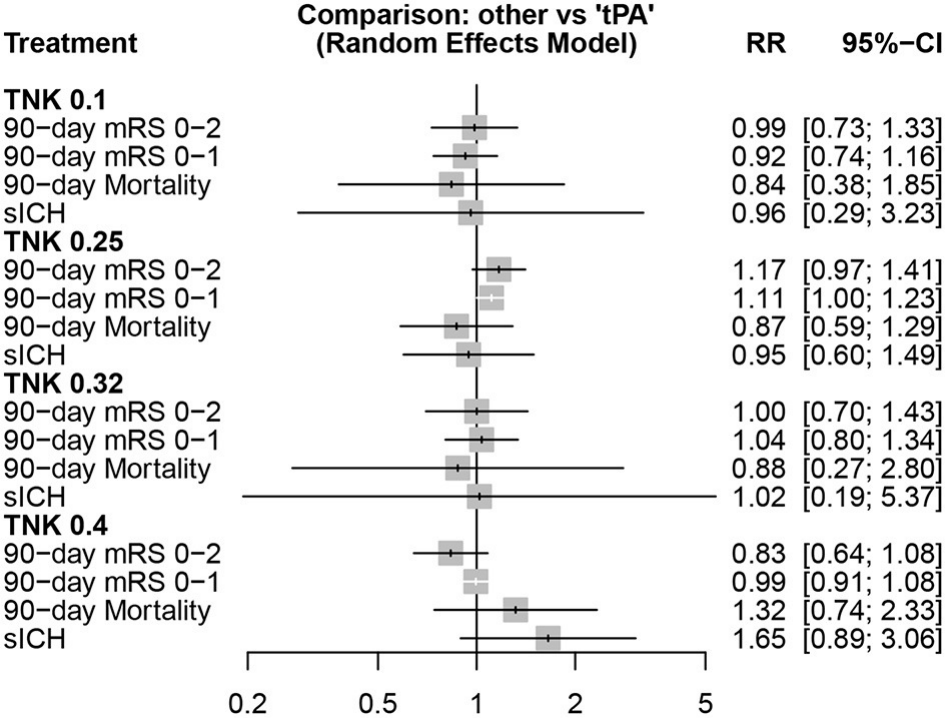
Different doses of tenecteplase (TNK) vs. alteplase (tPA).

Comparisons of different tenecteplase doses to alteplase, in different outcomes, are shown in Figure 5.

The highest rate of functional independence (mRS 0–2) was observed with tenecteplase 0.25 mg/kg (*P*-score = 0.904), followed by tenecteplase 0.32 mg/kg (*P*-score = 0.505), alteplase (*P*-score = 0.500), tenecteplase 0.1 mg/kg (*P*-score = 0.462), and tenecteplase 0.4 mg/kg (*P*-score = 0.129), respectively. However, there was significant heterogeneity/inconsistency among included studies (I^2^ = 69.3%, *P*-value = 0.003). Publication bias was identified by Begg-Mazumdar test (*P*-value = 0.037), but not with other two tests (Supplementary Figure 3).

For mRS 0–1, tenecteplase 0.25 mg/kg (*P*-score = 0.893) showed the highest rates, followed by tenecteplase 0.32 mg/kg (*P*-score = 0.613), alteplase (*P*-score = 0.494), tenecteplase 0.1 mg/kg (*P*-score = 0.302), and tenecteplase 0.4 mg/kg (*P*-score = 0.198), respectively. There was no heterogeneity/inconsistency among included studies (I^2^ = 31.7%, *P*-value = 0.146), and no publication bias was detected by any of the tests employed (Supplementary Figure 4). In pairwise comparisons, tenecteplase 0.4 mg/kg yielded significantly lower rates of mRS 0-2 compared to tenecteplase 0.25 mg/kg (RR = 0.71; 95% CI = 0.52–0.98). For all other pairwise comparisons, all tenecteplase dosing regimens were comparable to alteplase and among each other for both mRS 0–1 and mRS 0–2 (Table 2A).

**Table 2A:** Network meta-analysis of different treatments and functional outcomes—mRS 0–2 (lower part) and mRS 0–1 (upper part) rates.

**TNK 0.4**	**0.85 (0.58 to 1.25)**	**0.78 (0.60 to 1.01)**	**0.96 (0.69 to 1.34)**	**0.90 (0.73 to 1.10)**
0.83 (0.54 to 1.30)	**TNK 0.32**	0.91 (0.66 to 1.27)	1.13 (0.79 to 1.61)	1.05 (0.76 to 1.46)
**0.71 (0.52 to 0.98)**	0.86 (0.60 to 1.22)	**TNK 0.25**	1.23 (0.95 to 1.61)	1.15 (0.98 to 1.35)
0.85 (0.57 to 1.26)	1.02 (0.69 to 1.49)	1.19 (0.89 to 1.59)	**TNK 0.1**	0.93 (0.71 to 1.22)
0.83 (0.64 to 1.08)	1.00 (0.70 to 1.43)	1.17 (0.97 to 1.41)	0.99 (0.73 to 1.33)	**tPA**

mRS, Modified rankle scale; TNK, tenecteplase; tPA, alteplase. Comparisons should be read from left to right. RR above 1 favors the row-defining category (higher rates of functional independence); Significant results are in bold font.

For mortality, the lowest mortality rates were observed with tenecteplase 0.25 mg/kg (*P*-score = 0.654), followed by tenecteplase 0.1 mg/kg (*P*-score = 0.639), tenecteplase 0.32 mg/kg (*P*-score = 0.571), alteplase (*P*-score = 0.453), and tenecteplase 0.4 mg/kg (*P*-score = 0.183), respectively. There was no heterogeneity/inconsistency across studies (I^2^ = 30.9%, *P*-value = 0.153), and no publication bias was detected (Supplementary Figure 5). Results were similar for sICH, where tenecteplase 0.25 mg/kg (*P*-score = 0.643) had the lowest rates, followed by tenecteplase 0.1 mg/kg (*P*-score = 0.584), alteplase (*P*-score = 0.583), tenecteplase 0.32 mg/kg (*P*-score = 0.532), and tenecteplase 0.4 mg/kg (*P*-score = 0.157), respectively. There was no heterogeneity heterogeneity/inconsistency among included studies (I^2^= 0.0%, *P*-value = 0.502), and no publication bias was detected (Supplementary Figure 6). On assessment of mortality and sICH, pairwise comparisons did not show any significant differences between all tenecteplase dosing regimens and alteplase or in comparison tenecteplase doses to each other (Table 2B).

**Table 2B:** Network meta-analysis of different treatments and functional outcomes—Mortality (lower part) and sICH (upper part) rates.

**TNK 0.4**	**1.62 (0.28 to 9.38)**	**1.75 (0.84 to 3.63)**	**1.72 (0.46 to 6.51)**	**1.65 (0.89 to 3.06)**
1.50 (0.41 to 5.44)	**TNK 0.32**	1.08 (0.20 to 5.86)	1.06 (0.23 to 4.88)	1.02 (0.19 to 5.37)
1.52 (0.77 to 2.97)	1.01 (0.30 to 3.36)	**TNK 0.25**	0.98 (0.28 to 3.45)	0.95 (0.60 to 1.49)
1.57 (0.60 to 4.08)	1.05 (0.32 to 3.42)	1.04 (0.45 to 2.40)	**TNK 0.1**	0.96 (0.29 to 3.23)
1.32 (0.74 to 2.33)	0.88 (0.27 to 2.80)	0.87 (0.59 to 1.29)	0.84 (0.38 to 1.85)	**tPA**

sICH, symptomatic intracranial hemorrhage, TNK, tenecteplase; tPA, alteplase. Comparisons should be read from left to right. RR below 1 favors the row-defining category (lower mortality/sICH rates); Significant results are in bold font.

## Discussion

In this systematic review and meta-analysis of nine RCTs, we found that tenecteplase has similar safety and efficacy to alteplase for the treatment of AIS. Our pooled analysis results indicate that there was no difference in clinical outcomes (mRS 0–1 and mRS 0–2), sICH or mortality between tenecteplase and alteplase. Additionally, we found that similar outcomes were present with tenecteplase doses of 0.1, 0.25, 0.32, and 0.4 mg/kg. Our findings support the use of tenecteplase for thrombolytic treatment of AIS.

Our findings are consistent with previous studies that have examined the two thrombolytics. Nearly all RCTs in our analysis reported results that showed no statistically significant difference between tenecteplase and alteplase. The NOR-TEST-2, part A reported by Kvistad et al. was the only trial included in our analysis that reported worse outcomes with tenecteplase compared to alteplase. This trial found statistically significant higher rates of mortality and sICH along with lower rates of mRS 0–1 and mRS 0–2 with use of tenecteplase as compared to alteplase for patients with moderate or severe stroke (NIHSS ≥6). Kvistad et al. hypothesized that these results were due to a high dose of tenecteplase (0.4 mg/kg). To further investigate this, we conducted an analysis comparing the doses of tenecteplase, and found that there were no significant differences in safety or efficacy endpoints among different doses. Additionally, when Kvisted at al. data was pooled with other studies that used a dose of 0.4 mg/kg of tenecteplase, any statistically significant differences between tenecteplase and alteplase were no longer present for mRS 0–1, mRS 0–2, mortality, and sICH. However, it is important to keep in mind that other trials that tested the 0.4 mg/dose included patients with lower stroke severity (for instance, the median NIHSS in the first NOR-TEST trial reported by Logallo et al. was 4) (11). It is also important to note that in the EXTEND-IA TNK trial reported by Campbell et al., tenecteplase 0.25 mg/kg was superior to alteplase for patients presenting with large vessel occlusion who were eligible for mechanical thrombectomy, both in terms of rates of reperfusion and 90-day functional outcome (12). Meanwhile, the EXTEND-IA TNK part 2 compared tenecteplase 0.4 mg/kg vs. tenecteplase 0.25 mg/kg in patients with large vessel occlusion and found no efficacy advantage and a trend toward higher risk of sICH with the higher dose (23). Thus, available evidence supports the use of tenecteplase at a dose of 0.25 mg/kg.

Our study has limitations. We did not have access to patient-level data from individual studies which limited the analysis we were able to perform. The trials included in our meta-analysis tested different doses of tenecteplase. Yet, the 0.25 mg/kg dose was used most commonly. While we performed a comparison among the three tenecteplase doses (0.1, 0.25, 0.32, and 0.4 mg/kg) and found no differences in safety or efficacy endpoints, the data are stronger for the 0.25 mg/kg dose. Additionally, only one study tested a dose of tenecteplase of 0.32 mg/kg (13). Of the nine RCTs in our analysis, six studies used a treatment window of < 4.5 h, Parsons et al. used a treatment window of < 6 h, and Haley et al. and Li et al. used a treatment window of < 3 h. The variation in treatment windows is a potential source of bias and heterogeneity that we could not control for. Also, different trials used different definitions of sICH. Finally, we were unable to compare rates of arterial recanalization and other variables because the majority of included RCTs did not report the data or reported data in a heterogenous manner, highlighting the need for common data elements among RCTs. Our study's chief strength is that we only included in our meta-analysis RCTs comparing both thrombolytic agents head-to-head, allowing us to achieve the highest level of evidence possible (24).

## Conclusions

In this meta-analysis of nine RCTs of patients treated for AIS, we found comparable rates of favorable functional outcomes (mRS 0–1 and mRS 0–2) at 90 days, sICH and mortality between tenecteplase and alteplase. Our study supports the use of tenecteplase as a reasonable treatment for AIS, particularly considering its practical administration advantages over alteplase.

## Data availability statement

The original contributions presented in the study are included in the article/Supplementary material, further inquiries can be directed to the corresponding author.

## Ethics statement

Ethical review and approval was not required for the study on human participants in accordance with the local legislation and institutional requirements. Written informed consent for participation was not required for this study in accordance with the national legislation and the institutional requirements.

## Author contributions

All authors listed have made a substantial, direct, and intellectual contribution to the work and approved it for publication.
