# Supplementary material for: Tenecteplase vs. alteplase for treatment of acute ischemic stroke: A systematic review and meta-analysis of randomized trials
Source: Front Neurol. 2023 Jan 23;14:1102463. doi: 10.3389/fneur.2023.1102463 (PMC9900099; doi:10.3389/fneur.2023.1102463)
Supplement: Supplementary file 2 [file Data_Sheet_1.docx]

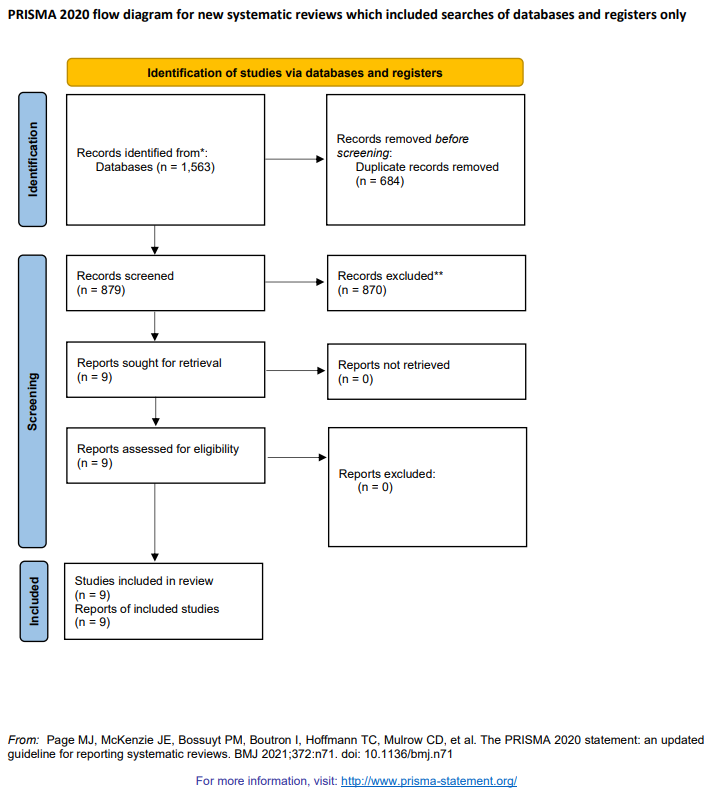


Supplementary Figure 1: PRISMA flow diagram detailing the literature search process


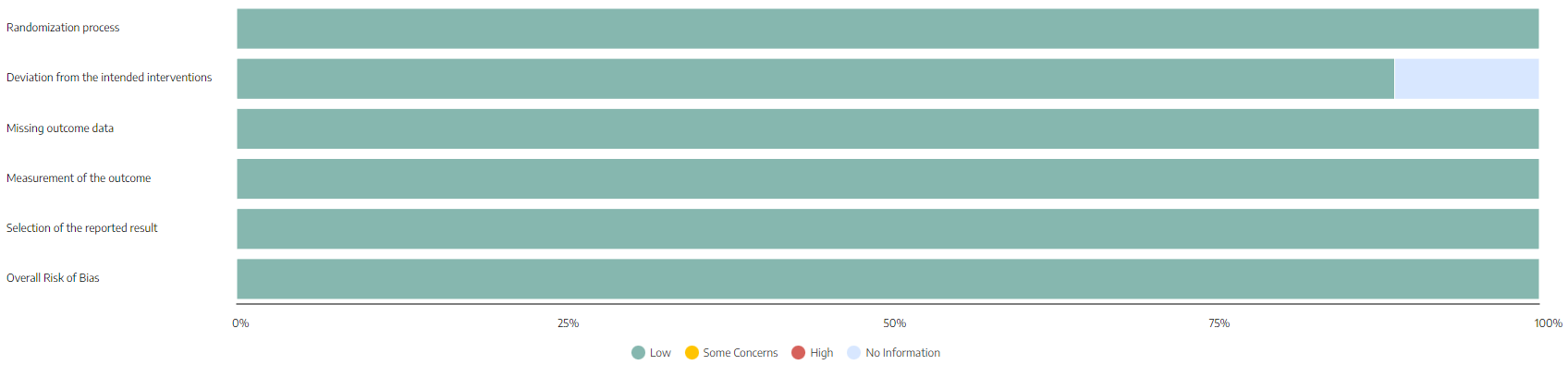


Supplementary Figure 2: Risk of bias domain distribution


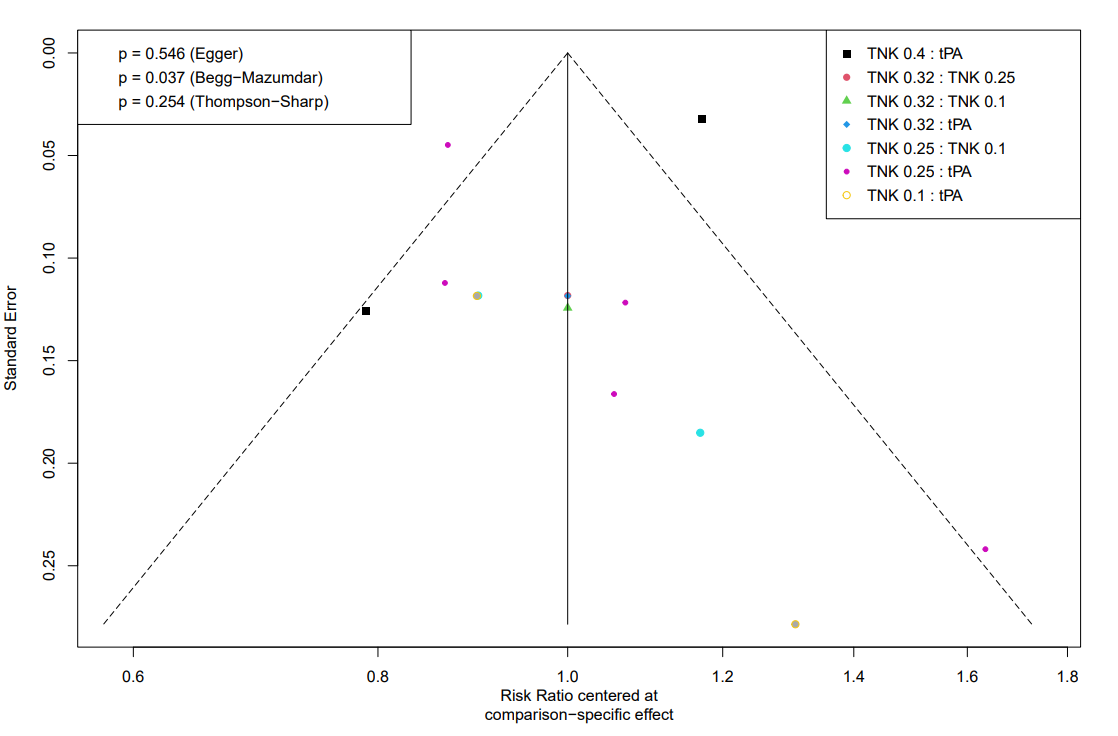


Supplementary Figure 3: Comparison-adjusted funnel plot for mRS 0-2 - with tests of publication bias


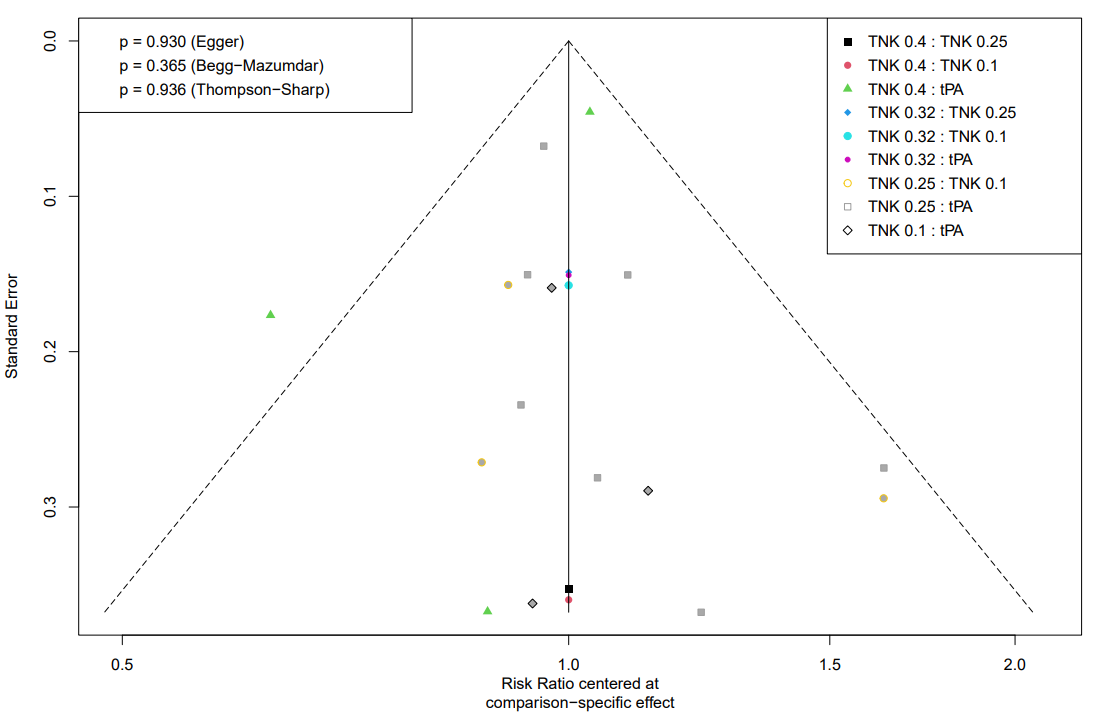


Supplementary Figure 4: Comparison-adjusted funnel plot for mRS 0-1 - with tests of publication bias


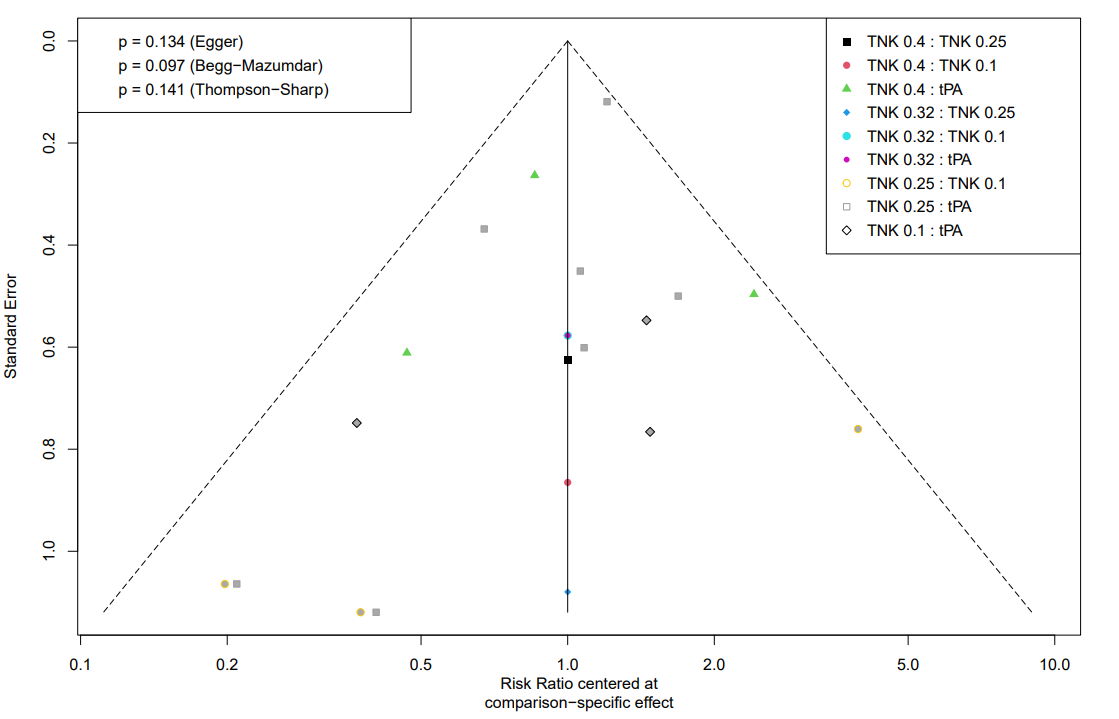


Supplementary Figure 5: Comparison-adjusted funnel plot for mortality - with tests of publication bias


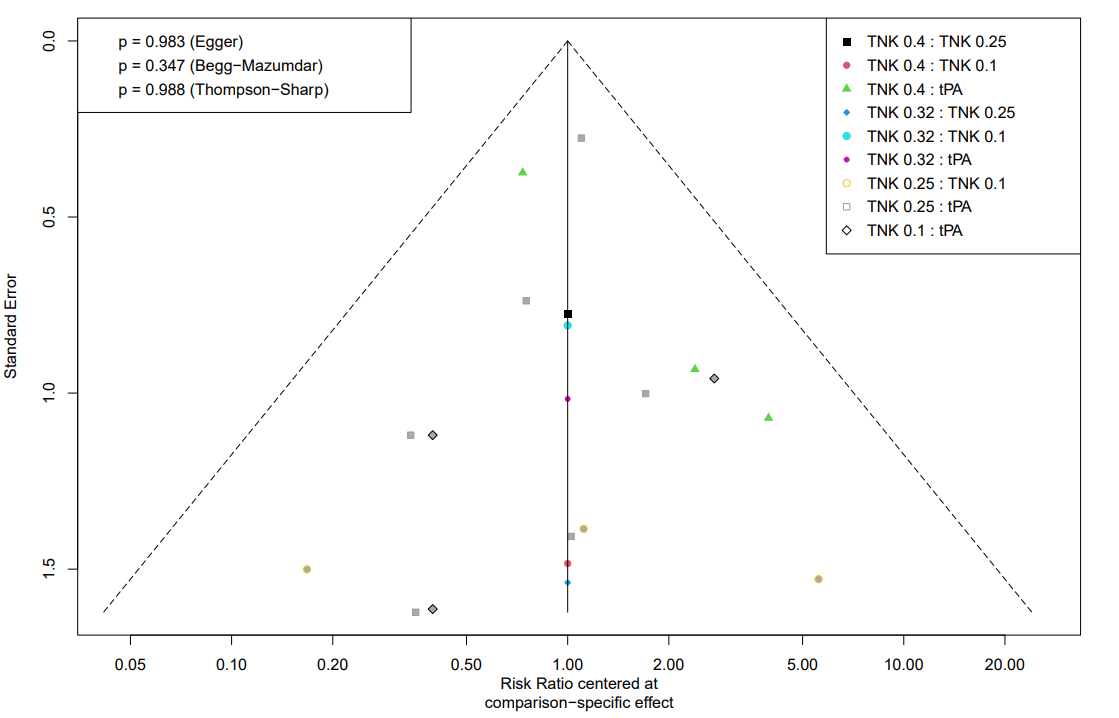


Supplementary Figure 6: Comparison-adjusted funnel plot for sICH - with tests of publication bias
